# Innovative LIDAR 3D Dynamic Measurement System to Estimate Fruit-Tree Leaf Area

**DOI:** 10.3390/s110605769

**Published:** 2011-05-27

**Authors:** Ricardo Sanz-Cortiella, Jordi Llorens-Calveras, Alexandre Escolà, Jaume Arnó-Satorra, Manel Ribes-Dasi, Joan Masip-Vilalta, Ferran Camp, Felip Gràcia-Aguilá, Francesc Solanelles-Batlle, Santiago Planas-DeMartí, Tomàs Pallejà-Cabré, Jordi Palacin-Roca, Eduard Gregorio-Lopez, Ignacio Del-Moral-Martínez, Joan R. Rosell-Polo

**Affiliations:** 1 Department of Agro-forestry Engineering, University of Lleida, Avinguda Rovira Roure 191, 25198 Lleida, Spain; E-Mails: aescola@eagrof.udl.cat (A.E.); jarno@eagrof.udl.cat (J.A.-S.); manelo@eagrof.udl.cat (M.R.-D.); jmv@eagrof.udl.cat (J.M.-V.) splanas@eagrof.udl.cat (S.P.-D.); egregorio@eagrof.udl.cat (E.G.-L); igdemo@eagrof.udl.cat (I.D.M.-M); jr.rosell@eagrof.udl.cat (J.R.R.-P.); 2 Department of AgriFood Engineering and Biotechnology, Polytechnic University of Catalonia, Campus del Baix Llobregat, edifici D4, Av. del Canal Olímpic, s/n 08860 Castelldefels, Spain; E-Mail: jordi.llorens.calveras@upc.edu; 3 Centre de Mecanització Agrària, Department of Agriculture, Livestock, Fisheries, Food and Natural Environment, Generalitat de Catalunya, 25197 Lleida, Spain; E-Mails: ferran.camp@gencat.cat (F.C.); felipj.gracia@gencat.net (F.G.-A.); fsolanelles@gencat.net (F.S.-B.); 4 Department of Computer Science and Industrial Engineering, University of Lleida, Av. Jaume II 69, 25197 Lleida, Spain; E-Mails: tpalleja@diei.udl.cat (T.P.-C.); palacin@diei.udl.cat (J.P.-R.)

**Keywords:** terrestrial laser scanner, LIDAR, 3D plant structure, fruit tree, leaf area

## Abstract

In this work, a LIDAR-based 3D Dynamic Measurement System is presented and evaluated for the geometric characterization of tree crops. Using this measurement system, trees were scanned from two opposing sides to obtain two three-dimensional point clouds. After registration of the point clouds, a simple and easily obtainable parameter is the number of impacts received by the scanned vegetation. The work in this study is based on the hypothesis of the existence of a linear relationship between the number of impacts of the LIDAR sensor laser beam on the vegetation and the tree leaf area. Tests performed under laboratory conditions using an ornamental tree and, subsequently, in a pear tree orchard demonstrate the correct operation of the measurement system presented in this paper. The results from both the laboratory and field tests confirm the initial hypothesis and the 3D Dynamic Measurement System is validated in field operation. This opens the door to new lines of research centred on the geometric characterization of tree crops in the field of agriculture and, more specifically, in precision fruit growing.

## 1. Introduction

The geometric characterization of tree orchards is a non-destructive precision activity, which entails measuring and acquiring precise knowledge of the geometry and structure of the trees [1]. It is both an important and complex task. Its importance lies in the fact that a wide range of agricultural activities (including the application of plant protection products, irrigation, fertilization, pruning, *etc.*) depend to a large extent on the structural and geometric properties of the visible part of the trees. Its complexity is due to the different elements which make up a tree, and the difficulty of measuring these elements. There are three basic reasons for this difficulty: (i) the large number of elements, (ii) the layout of a relatively small three-dimensional space which, from any point of view, will always have elements that are totally or partially hidden and (iii) the geometric complexity of all the elements.

The measurement and structural characterization of plants can be carried out remotely using a number of detection approaches. These include image analysis techniques, stereoscopic photography, analysis of the light spectrum and ultrasonic sensors [2]. There are some drawbacks to these systems. For example, image-based canopy measurements require elaborate algorithms and fast computational resources in order to operate in real-time. The angle of divergence of the ultrasonic waves limits the spatial resolution and accuracy of ultrasonic sensors [3]. Another approach involves the use of LIDAR (LIght Detection And Ranging) sensors, which can be airborne (satellites and aircraft) or ground-based. LIDAR systems can measure the distance between the sensor and the objects around it very quickly, enabling the construction of three-dimensional point clouds. Through the application of appropriate algorithms, these point clouds can be used to digitally reconstruct and describe the structure of trees with high levels of precision [2,4–11]. The capacity to quantify spatial variations, which is an important aspect of vegetation structure, is a significant advance over some previous methods. LIDAR systems can be used to quantify changes in canopy structure at various time scales. They can provide detailed assessment of canopy growth and allocation response to field experiments including fertilization, irrigation and plant protection. They can also be used as a tool for long-term studies of vegetation change. The measurements they take could be used to investigate interactions between structures and microclimates or structures and atmospheric exchanges [12]. Two-Dimensional Terrestrial Laser-LIDAR Scanners (2D TLS) make two-dimensional sweeps in just one measuring plane. The additional third dimension is obtained by moving the sensor in a perpendicular direction to the scanning plane [2,4,14]. Though 2D TLS systems are normally simpler and more affordable than 3D TLS systems they tend to be less accurate and it can be difficult to properly control the movement of the sensor when collecting the data [3]. The following three sections contain a brief review of the few studies that have been carried out using TLS systems with tree crops. They have been classified into three groups. The first group is of four studies, which used data supplied by 3D TLS tripod-mounted systems [6,10,11,15]. The second group is of various studies, which used data obtained with 2D TLS tractor-mounted systems [3,16–20]. In this second group, the data obtained from the two sides of the fruit tree rows were not registered into a single system of coordinates. The third group of studies also used data obtained with tractor-mounted 2D TLS systems. However, in this group, the data acquired from the two sides of the fruit tree rows were registered into a single system of coordinates [2,4].

### 1.1. Studies with 3D TLS Tripod-Mounted Systems

An Intelligent Laser Ranging and Imaging System (ILRIS-3D) sensor was used in [6] to characterize individual tree crowns at olive (*Olea europaea L.*) plantations in Cordoba, Spain. In addition to conventional 3D TLS tripod-mounted ILRIS-3D scans, the unit was mounted on a platform (12 m above ground) to provide nadir (top-down) observations of the olive crowns. The measurement system was evaluated and tested in the laboratory prior to its use in the field [15]. The objective of this research was to develop approaches to use ILRIS-3D data to retrieve structural information of an artificial tree in a controlled laboratory experiment. In another study, a 3D TLS tripod-mounted system provided the means to generate high-resolution volumetric measures [10]. Data collected from grapevine trunks and cordons were used to study the accuracy of wood volume derived from laser scanning compared with volume derived from analogue measurements. In [11] an attempt was made to obtain a precise 3D image of a tomato canopy using a portable high-resolution scanning LIDAR. The tomato canopy was scanned from three positions surrounding it. The point cloud data of the canopy were acquired and registered. Afterwards, points corresponding to leaves were extracted and converted into polygons.

### 1.2. Studies with 2D TLS Tractor-Mounted Systems, Without Registering of Data

A review was undertaken in [16] of spray volume deposition models that have been developed to enable the adjustment of pesticide output from an axial fan sprayer to suit different apple orchards. A 2D TLS tractor-mounted system was used to record orchard structural detail. In [17] a laser scanning system and corresponding algorithms were developed for potential use in estimating tree canopy height, width and volume. Spatial resolution of the system was estimated to be smaller than 6.0 cm (horizontal) × 1.9 cm (vertical). In [18] a laser scanning system was used to quantify foliage density of citrus trees. The density estimations were based on the laser sensor-canopy distance measurements and a canopy boundary-smoothing algorithm. Ten citrus trees with different foliage densities were scanned by the laser system. The calculated results were then compared with the corresponding visual assessments of tree densities. The relationship between tree volume and foliage was analyzed in [19]. In this work, tree volume was estimated with a terrestrial LIDAR system and subsequently used to estimate the total leaf area. In [3] the sensitivity of tree volume estimates relative to different error sources in the estimated spatial trajectory of the LIDAR system was analyzed. Tests with pear trees demonstrated that estimation of the canopy volume is very sensitive to errors in the determination of the distance from the LIDAR system to the centre of the trees. In [20], a 2D TLS tractor-mounted system was used to scan citrus fruit trees from one side of the rows. The objective of the study was to develop a laser scanner based system and associated algorithms for the measurement of tree geometric characteristics such as tree canopy, height, width, surface area, and volume. The laser scanner based measurement system developed in the study demonstrated an ability to estimate these parameters with a relatively good accuracy. However, measurement errors may increase for asymmetrically shaped trees canopies.

### 1.3. Studies with 2D TLS Tractor-Mounted Systems, With Registering of Data

Two papers [2,4] have already been published which evaluated in actual fruit tree orchards the measurement system presented in this paper. In [2] the measurement system was tested in fruit tree orchards, obtaining a single cloud of points from the registration of the clouds obtained from each side of the fruit tree rows. The registration process is explained in detail in this paper. In [4] a good correlation was found between LIDAR-based volume estimations of tree-row plantations and manual volume measurements.

### 1.4. Hypothesis and Objectives

In this work, a LIDAR-based 3D Dynamic Measurement System is presented and evaluated for the geometric characterization of tree crops. Using this measurement system, trees were scanned from two opposing sides to obtain two three-dimensional point clouds. After registration of the point clouds, a simple and easily obtainable parameter is the number of impacts received by the scanned vegetation. Given that their main function is photosynthesis, the distribution and position of leaves is clearly related to the availability of light. For this reason, the preferred position of leaves is normally in the outer part of the crown. With these premises, this work is based on the hypothesis that there may exist a linear relationship between the number of impacts of the LIDAR sensor laser beam and the tree leaf area.

The specific objectives that are considered in this work are as follows:

- To evaluate of a 3D Dynamic Measurement System based on the 2D-TLS SICK LMS200 LIDAR sensor (SICK AG.) in dynamic conditions and at small laboratory scale.
- To study the relationship between the number of impacts of the LIDAR laser beam on the vegetation and the leaf area of that vegetation. This study was first conducted under laboratory conditions using an ornamental tree and, subsequently, in a commercial pear tree orchard.

## 2. Materials and Methods

Section 2.1 describes the LIDAR sensor used in the laboratory and field tests. Section 2.2 details the specific materials and methods used in the laboratory work. Section 2.3 details the specific materials and methods of the field tests conducted in a pear tree orchard.

### 2.1. Description of the LIDAR Sensor

**Figure 1.**
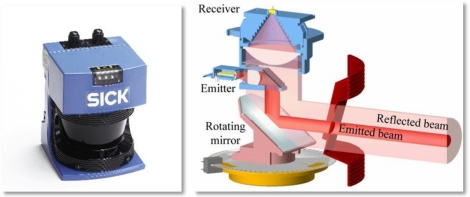
LMS200 laser sensor (SICK AG) and its principal internal components.

The terrestrial SICK LMS200 LIDAR sensor was chosen for this study (Figure 1). This sensor is widely used within the industry for such varied applications as: (i) surveillance systems; (ii) counting and measuring systems; (iii) anti-collision systems; (iv) artificial vision for robots and self-guided equipment. It is a 2D sensor, which only scans in one measuring plane. This makes its cost very low compared to 3D TLS sensors which generally make more precise sweeps of three-dimensional spaces, and with a greater distance range, than the LMS200.

The LMS200 is an eye safe (Class 1), time-of-flight laser sensor that emits at a wavelength of 905 nm (near infrared). Collaborative targets with specific reflectance features are not necessary and no lighting is required other than that provided by the emitted laser beam [21]. The sensor gives the estimations in a polar form, providing a distance and its angle for each measuring point. Within the range from 0 to 8 m, the distance resolution is equal to 1 mm and the standard deviation is ±1.5 cm. The maximum angular range is 0°–180° but smaller ranges can be configured. The beam directions of 0° and 180° are both vertical, pointing upwards and downwards, respectively. The angular resolution can be configured by the user with a choice of three possible values: 1°, 0.5° and 0.25°. The first two values were used in this test. The angular resolution of 0.25° was not used because the angular range is then limited to a maximum of 100°. Using the maximum angular range (0°–180°) and the selected angular resolution, the following information was obtained with each scan.

- A total of 181 distance measurements using an angular resolution of 1°. These were obtained from a single complete rotation of the mirror (Figure 1).
- A total of 361 distance measurements using an angular resolution of 0.5°. These were obtained from two complete rotations of the mirror. Obtaining measurements with 0.5° angular resolution requires twice the amount of time compared with a 1° angular resolution.

**Figure 2.**
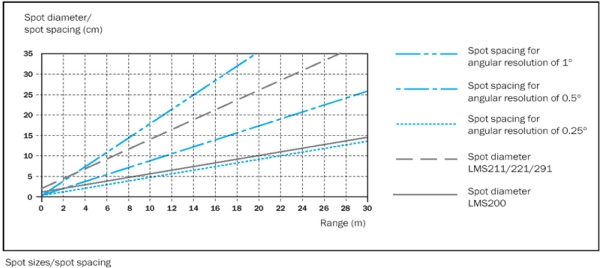
Diameter of the laser beam cross-section of various models of the SICK LMS series and the separation between beams as a function of the angular resolution and distance to the sensor [21].

The number of measurements per second was the same with both angular resolutions. The RS-232 data transfer protocol was used between the computer and the sensor at a speed of 38,400 bits per second. It was verified that at this communication speed the sensor performs 1,700 distance measurements per second. Figure 2 shows the laser beam section diameter (spot diameter) of various models of the LMS series and the separation between beams (spot spacing) as a function of the angular resolution and distance to the sensor.

**Figure 3.**
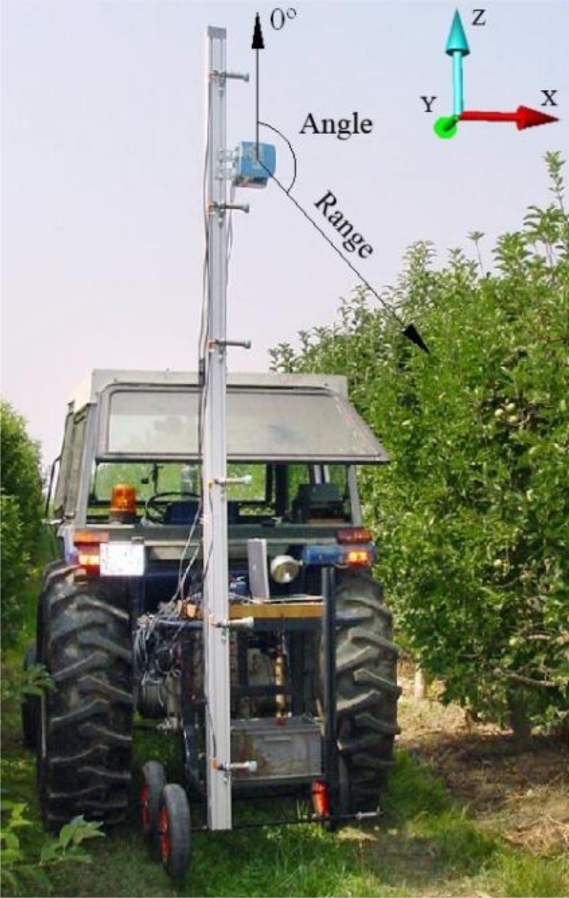
The impact points of the laser beam are determined in polar coordinates. The direction of the laser beam with an angle of 0° is vertical and upward pointing. The Cartesian coordinate system (X,Y,Z) used in the field and laboratory is also shown.

Figure 3 shows the sensor and the coordinate system adopted in the field and laboratory. The XY plane is parallel to the ground, the positive direction of the Y-axis represents the forward motion of the sensor and the positive direction of the Z-axis is vertical up.

The direction of the Y-axis is a straight-line trajectory, which coincides with the forward motion of the LIDAR system. It is very important for the LIDAR system to travel at constant speed and follow an accurate straight-line path. In order to determine the value of the Y-coordinate of each single scan (slice), the developed software stores the time (in milliseconds) between slices. Since the LIDAR system is moving in a straight-line and at constant speed, the transformation of time into distance is direct.

### 2.2. Materials and Methods Used in the Laboratory Tests

It was decided to set up a dynamic system in the laboratory before testing the LIDAR system in the field in fruit tree orchards [2,4]. The objective was to observe the performance of the LIDAR measurement system working with plant material under controlled conditions and with perfect straight-line motion at different constant speeds. The following material was used for these tests: A mechanised multi-purpose test rail, a SICK LMS200 sensor, a computer, an ornamental tree (*Ficus benjamina*), a metallic reference structure and a dynamic leaf planimeter.

**Figure 4.**
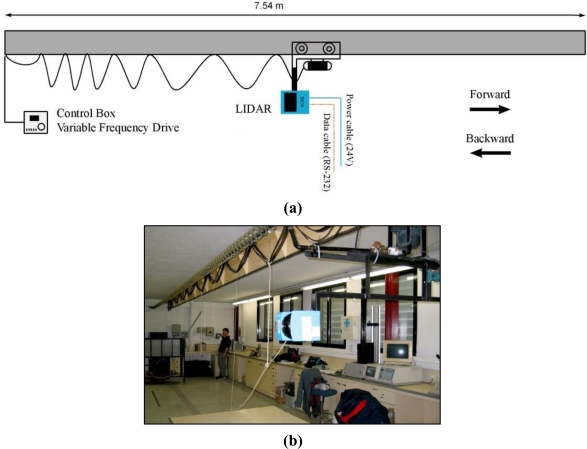
**(a)** Assembly layout of the LMS200 sensor on the motorised rail. **(b)** Photograph of the test rail.

The motorised multi-purpose test rail was 7.54 m long and allowed constant speeds of up to 2.3 km/h. This rail had a mobile structure, which could move in both directions and was driven by an AC motor, which is controlled by a variable-frequency drive. The LMS200 sensor was mounted on this structure (Figure 4). After mounting, the distance between sensor and floor was 176 cm.

**Figure 5.**
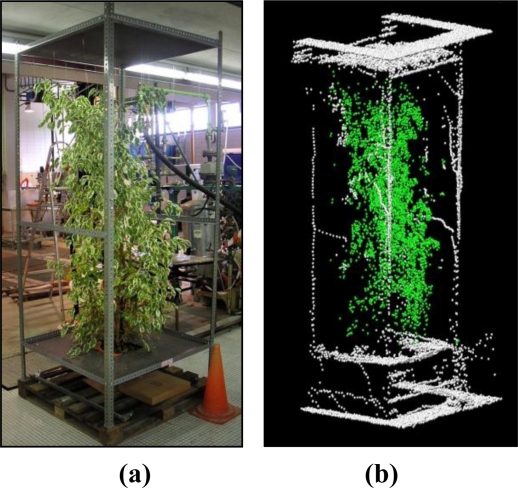
**(a)** Photograph of the *Ficus benjamina* inside the reference structure. **(b)** Graphic representation of the point cloud obtained with the LIDAR system.

Since this study was carried out during the winter period an evergreen tree was used. A 2 m tall *Ficus benjamina* was chosen for this study. The tree was placed inside a reference structure made from metal slotted angle sections (Figure 5).

**Figure 6.**
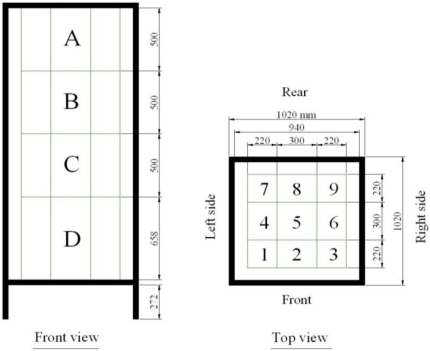
Front and top view of the internal division of the reference structure, together with the dimensions in mm and nomenclature.

The interior of this structure was sub-divided into rectangular prisms by using a nylon thread framework (Figure 6). This framework was put into position after the *Ficus* had been placed in the structure. The number of subdivisions (boxes) totalled 36, corresponding to 4 layers of height (A, B, C and D) and 9 rectangular boxes for each layer. The reference structure was placed together with the *Ficus* on a wooden pallet [Figure 5(a)] to facilitate its rotation.

The minimum distance between the LIDAR and the front of the reference structure was 500 mm. The minimum distance between the LIDAR and the mid-plane of the tree was 1,000 mm. The reference structure was placed approximately half way along the mechanised rail. In this way there was plenty of time for the speed at which the sensor was moving to stabilise before passing in front of the *Ficus*. Data acquisition with the LIDAR system was performed with different configurations, according to the following variables.

- Angular resolution of the LMS200 sensor: The sensor was set to angular resolutions of 1° and 0.5°.
- Travelling speed: The sensor was made to advance along the rail at 3 different speeds; 0.5, 1 and 1.5 km/h.
- Orientation of the structure: Front and rear view scans of the *Ficus* were performed.

Using different combinations of these three variables, various LIDAR scans of the *Ficus* and the reference structure were performed. The mobile structure and mounted sensor travelled in a forward direction for all the scans so as not to introduce any further variability. The whole structure (reference structure + *Ficus*) was rotated 180° for the rear view scan.

**Figure 7.**
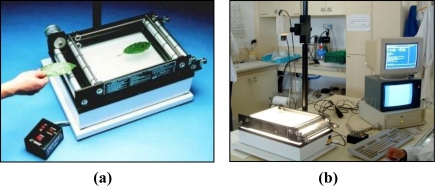
**(a)** Detailed Photograph of the Area Measurement System-Conveyor Belt Unit (Delta-T Devices Ltd.). **(b)** Photograph of all the equipment in the laboratory.

All the leaves of the *Ficus* were removed after the scanning work had been completed. The surface area was then measured of each leaf of each of the 36 subdivisions of the reference structure. The planimeter used for this purpose was an Area Measurement System-Conveyor Belt Unit (Delta-T Devices Ltd). This equipment consists of two different basic parts: A conveyor belt [Figure 7(a)] and an image analysis system [Figure 7(b)].

The following procedure was used to determine the number of laser beam impacts in each of the 36 reference structure subdivisions:

1. LIDAR scanning of the *Ficus* and the reference structure. The resulting file contained the coordinates of all the laser beam impact points.
2. Visualisation of the three-dimensional point cloud using the AUTOCAD 2004 software (Autodesk, Inc.).
3. Visual localization and subsequent numerical determination of the coordinates of the reference point located at the base of the reference structure.
4. Running of the post-processing software to calculate the impacts in each subdivision.

### 2.3. Materials and Methods Used in the Field Tests

**Figure 8.**
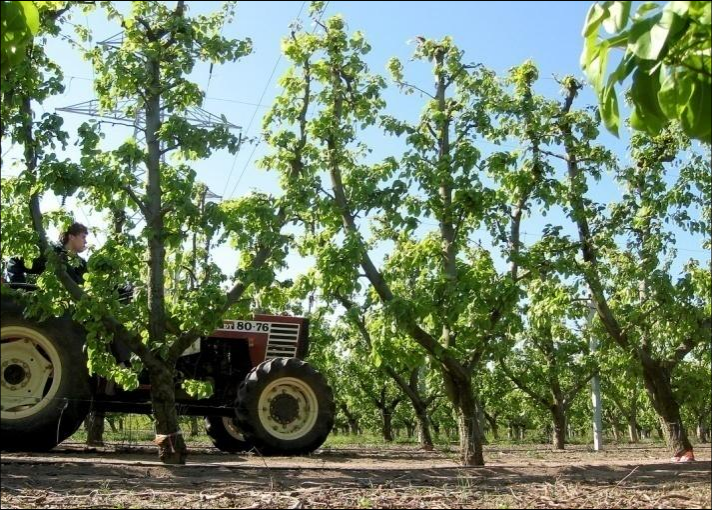
Photograph of Section 1 pear tree vegetation (*Pyrus communis* L. cv Blanquilla), Lleida, Spain, April 18.

After the winter time laboratory tests, the LIDAR measurement system was mounted onto a tractor and tested in the field to validate the initial hypothesis. These tests were conducted in a commercial pear tree orchard (*Pyrus communis* L. cv Blanquilla) in Lleida (Spain) (Figure 8).

**Figure 9.**
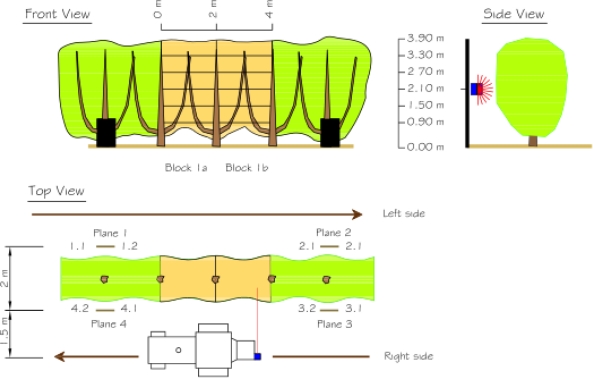
Schematic description of the tests performed in a pear tree orchard (*Pyrus communis* L. cv Blanquilla) in Lleida, Spain.

Figure 9 is a schematic description of the test. Four different 4 m long sections of vegetation were scanned using the LIDAR measurement system. Each section was scanned on a different date to ensure varying degrees of vegetation growth. Each section was subdivided lengthwise into two blocks of 2 m (distance between trunks) and vertically into six layers. The scanning process involved the displacement of the measurement system along the left-hand and right-hand sides of the section under study. The distance between the sensor and the ground was 2.1 m. An angular resolution of 1° was used. The distance between the sensor and the mid-plane of the tree row was 2.5 m. This distance enabled the use of a lower angular range (50°–160°) than the maximum permitted range (0°–180°). In this way it was possible to read more ‘useful’ points per second, eliminating the upper and lower regions (sky and ground), which contained no information about the vegetation. It was verified that with this configuration the sensor made 1,700 distance measurements per second, the same as in the laboratory tests.

The two scans were subsequently registered into a single point cloud. To ensure the correct registration of the two scans, the tractor was displaced in a straight-line path at a constant speed of 1 km/h. It should be mentioned that the degree of accuracy in this respect was, logically, less than that of the laboratory tests. Four reference planes were also used, two on each side, to facilitate the correct registration of the scans (Figure 9). Details of the complete registration process can be found in [2]. When the LIDAR scans had been concluded, the leaves were removed from each subdivision to find the distribution of the leaf surface area and relate it to the point cloud that had been obtained.

## 3. Results

In this section, the results obtained from the laboratory tests using an ornamental tree (*Ficus*) and the results of the field tests in a pear tree orchard are shown separately.

### 3.1. Results of the Laboratory Tests

Table 1 shows the 12 scans of the *Ficus* performed with the LIDAR system. It can be observed that the real forward speeds of the LIDAR system (column 4) are not exactly 0.5, 1, and 1.5 km/h. However, since the differences are small, these values will be used to facilitate the reading of this paper.

**Figure 10.**
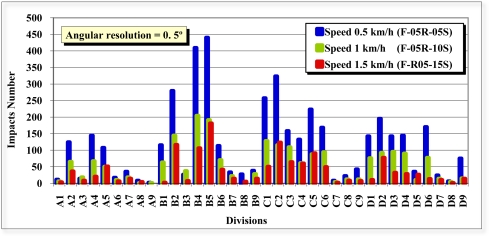
Impacts of scans F-05A-05S, F-05A-10S and F-05A-15S with angular resolution of 0.5°, and respective speeds of 0.5, 1.0 and 1.5 km/h.

**Figure 11.**
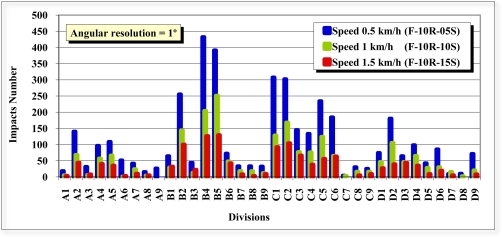
Impacts of scans F-10A-05S, F-10A-10S and F-10A-15S with angular resolution of 1°, and respective speeds of 0.5, 1.0 and 1.5 km/h.

In the front view scans (1–6), the effect was studied of the angular resolution (1°, 0.5°) and speed (0.5, 1 and 1.5 km/h) on the distribution of the laser beam impacts on the test tree. It can be observed in Figures 10 and 11 that, as we expected, the density of the laser beam impacts increased as the speed of the LIDAR was reduced.

The number of impacts was approximately inversely proportional to the speed. The peaks of highest number of impacts for the six scans of different configuration appeared in the same divisions (boxes). This was also expected. The divisions with the lowest number of impacts were, in this case, the rear divisions (7, 8 and 9), since the elements in them (leaves and branches) were concealed by the leaves and branches of the front (1, 2 and 3) and intermediate divisions (4, 5 and 6) (Figure 6).

With respect to the number of impacts, it should be remembered that for each scan 181 distances are obtained with an angular resolution of 1° and 361 distances with an angular resolution of 0.5°. However, in the latter case twice the amount of time is required. Therefore, with an angular resolution of 0.5°, and at the same speed of advance, the vertical resolution (V) is doubled and the horizontal resolution (H) halved in the displacement direction of the LIDAR (Y axis). It is not the density of the points which changes, but their distribution. If Figure 10 is compared with Figure 11, it can be observed that the number of impacts with angular resolutions of 0.5° and 1° are very similar in layers A, B and C for the three speeds of advance. However, it can be observed in layer D that more impacts occurred with an angular resolution of 0.5° than with a resolution of 1°. The D layer is the lowest layer of the tree, the furthest from the sensor and the most difficult to see/scan due to the proximity between the LIDAR and the tree. The reasons for the difference in the number of impacts are the position of layer D with respect to the sensor and the different distribution of the impacts with 0.5° and 1° angular resolution.

A verification was performed to compare the real dimensions of the reference structure with the dimensions obtained with the LIDAR system in all the scans. According to the data obtained with the LIDAR system, and after measuring the total height and width of the reference structure at various points, it was observed that the differences with respect to the real dimensions were ±1.5 cm.

**Figure 12.**
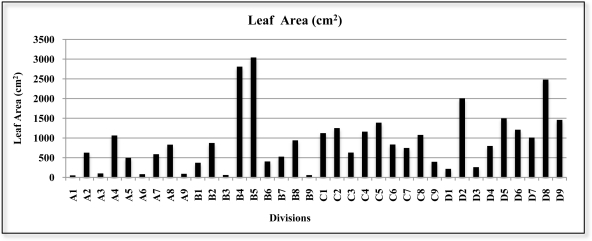
Leaf area of each of the 36 internal divisions of the reference structure.

After the *Ficus* had been scanned, the leaves were removed and the surface area of each leaf measured (1944 leaves). The results are shown in Figure 12 with the leaf area of each of the 36 internal divisions of the reference structure.

The front view F-05A-05S (Scan 1) and rear view R-05A-05S (Scan 7) scans were used to study the relationship between the number of impacts and the leaf area. Both scans were performed at a speed of 0.5 km/h and angular resolution of 0.5°. It was verified that, with this configuration, the resulting point mesh at the height of the sensor (beam angle of 90°) in the mid-plane of the *Ficus* was 2.9 cm in horizontal dimension (H) and 0.9 cm in vertical dimension (V).

**Figure 13.**
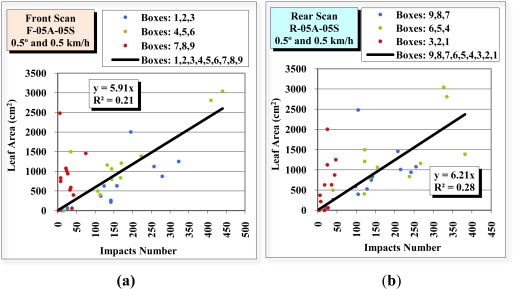
Scatter diagrams, regression lines and R^2^ of the relation between the number of impacts received in the 36 divisions (boxes) in the front view scan F-05A-05S **(a)** and rear view scan R-05A-05S **(b)**, and the leaf surface area of the same divisions.

Figure 13 shows the scatter diagram that results from correlation of the laser beam impacts to the leaf surface area of each division of the front view scan F-05A-05S and rear view scan R-05A-05S. The divisions that are furthest from the sensor (divisions 7, 8 and 9 for the front view scan and divisions 1, 2 and 3 for the rear view scan) are shown in red. The divisions closest to the sensor (divisions 1, 2 and 3 for the front view scan and divisions 9, 8 and 7 for the rear view scan) are shown in blue. Finally, the intermediate divisions (4, 5 and 6) are shown in green. The regression lines of all these points have low coefficient of determination (R^2^) values, 0.21 for the front view scan and 0.28 for the rear-view scan.

**Figure 14.**
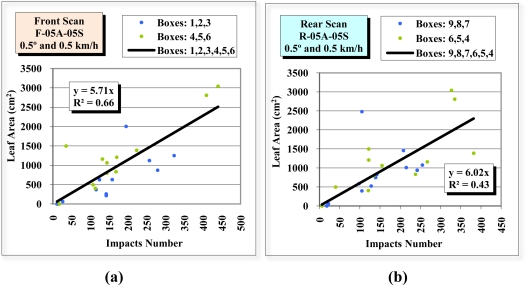
Scatter diagrams, regression lines and R^2^ of the relation between the number of impacts received in the 24 divisions (boxes) closest to the LIDAR sensor in the front view scan F-05A-05S **(a)** and rear-view scan R-05A-05S **(b)**, and the leaf surface area of the same divisions.

Figure 14 is constructed in exactly the same way as Figure 13, except that the information about the divisions furthest from the sensor (divisions 7, 8 and 9 in the front view scan and divisions 3, 2 and 1 in the rear view scan) are ignored. It can be seen that the R^2^ of the regression lines rise to values of 0.66 in the front view scan and 0.43 in the rear view scan.

**Figure 15.**
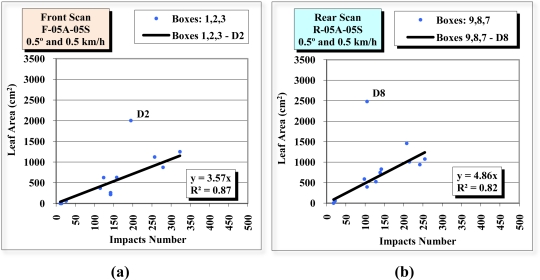
Scatter diagrams, regression lines and R^2^ of the relation between the number of impacts received in the 12 divisions (boxes) closest to the LIDAR sensor in the front view scan F-05A-05S **(a)** and rear view scan R-05A-05S **(b)**, and the leaf surface area of the same divisions. When obtaining the front view and rear view regression lines, divisions D2 and D8, respectively, were discarded.

Figure 15 is constructed in exactly the same way as Figure 14, except that only information from the divisions closest to the sensor (divisions 1, 2 and 3 in the front view scan and divisions 9, 8 and 7 in the rear view scan) is included. The information from division D2 in the front view scan and from D8 in the rear view scan has also been ignored, as these are points considered to be outliers. By observing the position of the points and the R^2^ of the regression lines (0.87 for the front view scan and 0.82 for the rear view scan), a good relationship can be observed between the number of impacts and the leaf surface area of the divisions closest to the sensor.

As we expected, the divisions furthest from the sensor received a lower number of impacts since they were concealed by the vegetation (leaves and branches) of the divisions closest to the sensor. For this reason, if we proceed to discard the obtained data starting from the rear and moving forwards towards the sensor, the relationship between the number of impacts and leaf surface area improves.

**Figure 16.**
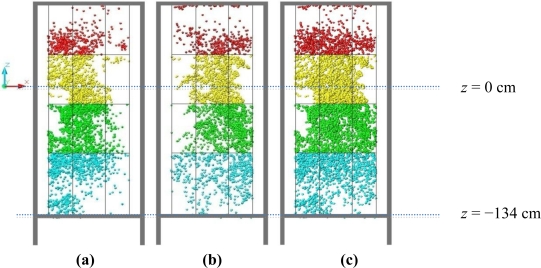
Left-hand side view of the reference structure and point cloud obtained from the rear view scan R-05A-05S **(a)**, front view scan F-05A-05S **(b)** and the two scans combined (**c**). The height position of the sensor is always 0 (*z* = 0).

**Figure 17.**
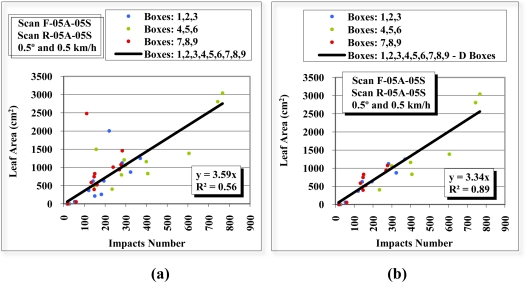
Scatter diagram, regression line and R^2^ of the relation between the number of impacts received in the 36 divisions (boxes), combining the front-view (F-05A-05S) and rear-view (R-05A-05S) scans, and the leaf surface area including layer “D” **(a)** and excluding layer “D” **(b)**.

After separate observation of the front and rear view scans, the results were combined (Figure 16) to examine the relationship between the total number of impacts of the two scans and the leaf surface area (Figure 17). The R^2^ of the regression line of all the points was 0.56 [Figure 17(a)], a clear improvement on the individual scan values of 0.21 and 0.28 (Figure 13). Figure 17(b) shows an R^2^ of 0.89. This was obtained after discarding the “D” divisions, the lowest and furthest layer from the sensor, as their values can be considered outliers.

The relative position between sensor and vegetation determines the quality of the measurements of the vegetation. For example, if the sensor is situated close to and in the upper part of the vegetation, the lower part of the vegetation will logically be concealed or hidden by the rest and, consequently, the laser beam will be unable to reach this lower area. This is what happened in the lower layer “D” of the *Ficus*, specifically in the divisions D2, D5, D8 and D9.

**Figure 18.**
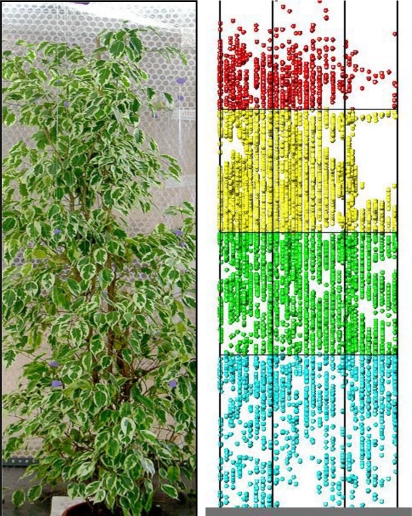
Front view photograph of the *Ficus* and, also taken from the front, view of the point cloud obtained after combining the front view (F-05A-05S) and rear view (R-05A-05S) scans obtained with the LIDAR system.

It can be observed in Figure 16 that the height of the sensor (*z* = 0) is positioned at some two thirds of the maximum height of the tree. It can be seen from observing the lower part of Figure 18 that there is reasonably abundant vegetation in the photograph of the *Ficus*. However, if we look at the blue points of layer “D” (and more specifically those that correspond to the central divisions of D2, D5 and D8) it can be seen that the impact density is not very high. This explains the difference in the values obtained from the divisions of the “D” layer. As the analysis of the data obtained from scans 8, 9, 10, 11 and 12 (Table 1) did not provide any additional information to the results as described above, it has not been included in this paper.

### 3.2. Results of the Field Tests

Having observed in the laboratory the good correlation between the number of impacts and the leaf area of a small *Ficus* the next step was to corroborate this relationship in a commercial pear tree orchard.

When designing this experiment special attention was given to the correct positioning of the LIDAR sensor. In order to guarantee the correct scanning of the vegetation, the sensor was positioned at an intermediate height of 2.1 m and an average distance from the vegetation of 2.5 m. This was done to avoid poor visualisation problems as occurred with the “D” layer of the *Ficus*. It was verified that the resulting point mesh at the height of the sensor (beam angle of 90°) in the mid-plane of the row was 1.9 cm in horizontal dimension (H) and 4.2 cm in vertical dimension (V).

Table 2 shows a list of the scans performed with the LIDAR measurement system in the pear tree orchard.

**Figure 19.**
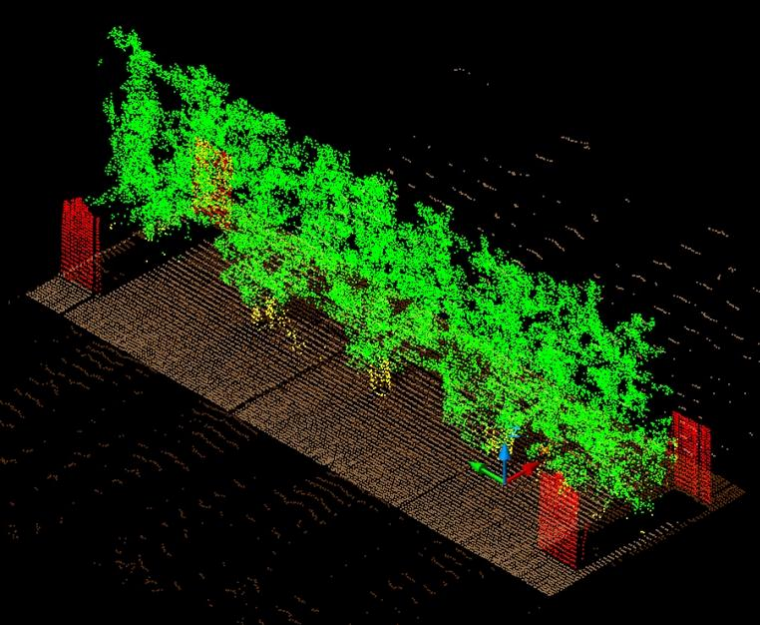
Point cloud obtained after registration of the scans of the left and right sides of Section 1.

When the scans of the right and left hand sides of each section had been concluded, they were then registered (Figure 19). The impacts obtained in each of the divisions of the four scanned sections were subsequently counted. All the divisions were then defoliated and the leaf area of each division calculated.

**Figure 20.**
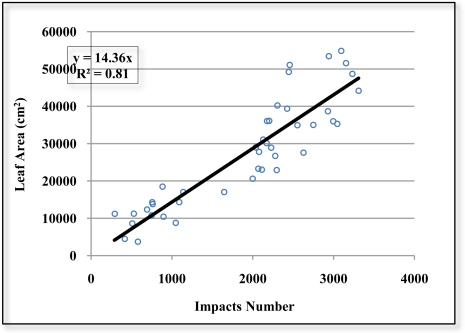
Scatter diagram, regression line and R^2^ of the relation between the number of impacts and leaf area (cm^2^) of all the divisions of the 8 defoliated blocks.

**Figure 21.**
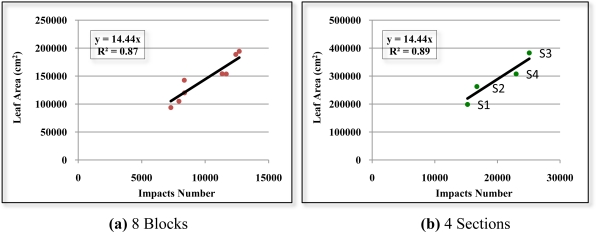
Scatter diagram, regression line and R^2^ of the relation between the number of impacts and leaf area (cm^2^) **(a)** ignoring the divisions by layer. **(b)** Also ignoring the “a” and “b” division between blocks

It can be observed in Figure 20 that there is a good correlation (R^2^ = 0.81) between the number of impacts and leaf area of all the divisions of the eight defoliated blocks. Figure 21(a) shows the correlation (R^2^ = 0.87) between the number of impacts and leaf area of the eight defoliated blocks when ignoring the division into layers. By grouping together the divisions, the variability that arises from working with small units of vegetation is reduced and the correlation is significantly improved. If the “a” and “b” division between blocks in the 4 scanned sections is also ignored, R^2^ reaches a value of 0.89 [Figure 21(b)].

The slope of the regression lines in the three cases is practically steady at 14.4. The vegetative state of the crop appears to have no influence on the relationship between the number of impacts and leaf area. It can also be observed in Figure 21(b) that section S3 has a higher leaf area despite being defoliated seven weeks before section S4. This may be due to the fact that a green pruning of the less productive branches was carried out in the orchard one week after the defoliation of S3

## 4. Discussion and Conclusions

### 4.1. Discussion

The results obtained in the laboratory with the *Ficus* and the results obtained in the field with the pear trees are difficult to compare for two reasons: Firstly, because the trees are very different and secondly, because different scanning meshes were used. With respect to the second problem, one solution that could enable work to be undertaken with slightly different meshes without affecting the results would be to use the equivalent surface area of an impact (H × V). That is, to replace the “number of impacts” variable with the “equivalent mesh surface area” variable. In this way, the mesh surface area can be related to the leaf area. This replacement of impacts with equivalent surface area needs to be analysed in greater detail in future studies.

Table 3 shows a summary of the results obtained in the laboratory tests (*Ficus*) and field tests with pear trees. In the second column, LA(cm^2^)/I, we have the equivalence between an impact (I) and the leaf area it represents (LA). Column 5, HV(cm^2^)/I, is the result of H × V. This value is the surface area of a grid of the point mesh, which represents or is equivalent to an impact. The results of 1.28 and 1.81 (LA/HV) for *Ficus* and pear trees, respectively, mean that for each unit of surface area perpendicular to the laser beam, we have a mean of 1.28 and 1.81 leaf area units.

### 4.2. Conclusions

The number and distribution of laser beam impacts on the tree used in the study (*Ficus benjamina*), as a function of the forward speed and angular resolution of the LIDAR system, are consistent with the technical specifications of the manufacturer and meet the pre-test expectations. This conclusion, together with the correct determination of the reference structure dimensions, confirms the proper operation of the LIDAR measurement system in motion and, moreover, of the developed software (data acquisition and post-processing modules).

The position of the sensor with respect to the vegetation is an important factor that needs to be taken into consideration as it affects considerably the viewing capabilities of the sensor and, consequently, the leaf area estimations.

Data capture from two opposing viewpoints considerably enhances the three-dimensional representation of the vegetation under study. Both the laboratory and field results confirm the initial hypothesis of the existence of a linear correlation between the number of laser beam impacts and the leaf area of the scanned vegetation. Replacement of the variable ‘number of impacts’ with the variable “impacted mesh surface area” is proposed to enable comparisons between tests using slightly different scanning meshes. This new measurement system opens the door to new lines of research related to the geometric characterization of tree crops in the field of precision agriculture.

### 4.3. Future Research

As the LIDAR sensor obtains its measurements in polar form, one problem that needs to be analysed is the influence of the shape of the mesh in terms of the type of vegetation being scanned [22].

Analysis is also required in future studies of the equivalence between an impact and the surface area of a grid. Further studies should also be carried out on the influence of the size of the cross section of the laser beam and on the phenomenon of mixed pixels [23]. This phenomenon appears when a laser beam impacts partially on two or more objects/targets.

Given the observation that in small divisions (Figure 20) there is more variability than in larger divisions (Figure 21), work is needed to determine the range of vegetation size suitable for leaf area estimation. To enable registrations that are more automated [2] and more accurate, consideration should be given to the incorporation of other sensors into the LIDAR measurement system. Sensors that may be suitable for incorporation include cm-accurate GPS systems and inertial measurement unit sensors, which detect angular changes of pitch, roll, and yaw.

In general terms, the continuation of the work undertaken in this study will involve specific studies using the LIDAR measurement system presented here. These studies will need to analyse a number of variables for different types of fruit tree of varying ages and different training and pruning methods.

## Figures and Tables

**Table 1.** Rear and front view scans of the *Ficus benjamina* specimen with angular resolutions of 0.5° and 1°, and approximate forward speeds of 0.5, 1 and 1.5 km/h.

**Scan**	**View**	**Angular resolution**	**Speed km/h**	**File**
1	Front	0.5°	0.494	F-05A-05S
2	Front	0.5°	1.000	F-05A-10S
3	Front	0.5°	1.520	F-05A-15S
4	Front	1.0°	0.494	F-10A -05S
5	Front	1.0°	1.000	F-10A-10S
6	Front	1.0°	1.520	F-10A-15S
7	Rear	0.5°	0.494	R-05A-05S
8	Rear	0.5°	1.000	R-05A-10S
9	Rear	0.5°	1.520	R-05A-15S
10	Rear	1.0°	0.494	R-10A-05S
11	Rear	1.0°	1.000	R-10A-10S
12	Rear	1.0°	1.520	R-10A-15S

**Table 2.** List of scans, performed on four different dates, of four sections of vegetation at a commercial pear tree orchard (*Pyrus communis* L. cv. Blanquilla) in Lleida, Spain.

**Scan**	**Date**	**Side**	**Section – Blocks**	**Scan File**
1	18-April	Left	S1 - (1a, 1b)	L1
2	18-April	Right	S1 - (1a, 1b)	R1
3	3-May	Left	S2 - (2a, 2b)	L2
4	3-May	Right	S2 - (2a, 2b)	R2
5	2-June	Left	S3 - (3a, 3b)	L3
6	2-June	Right	S3 - (3a, 3b)	R3
7	25- July	Left	S4 - (4a, 4b)	L4
8	25- July	Right	S4 - (4a, 4b)	R4

**Table 3.** Summary of the results obtained in the laboratory tests (*Ficus*) and in the field tests with pear trees. LA: leaf area. I: Impact. H, V: horizontal and vertical dimensions of a mesh grid in the mid-plane of the vegetation at the height of the LIDAR. HV = H × V.

**Mesh**					
	**LA(cm^2^)/I**	**H (cm)**	**V (cm)**	**HV(cm^2^)/I**	**LA/HV**
**Ficus**	3.34	2.9	0.9	2.61	1.28
**Pear trees**	14.44	1.9	4.2	7.98	1.81
